# Intracellular and extracellular factors of colorectal cancer liver metastasis: a pivotal perplex to be fully elucidated

**DOI:** 10.1186/s12935-022-02766-w

**Published:** 2022-11-08

**Authors:** Yaru Niu, Wenwei Yang, Haili Qian, Yongkun Sun

**Affiliations:** 1 Department of Medical Oncology, National Cancer Center/National Clinical Research Center for Cancer/Cancer Hospital, Chinese Academy of Medical Sciences and Peking Union Medical College, 17 Panjiayuan Nanli, Chaoyang District, Beijing, 100021 China; 2 National Cancer Center/National Clinical Research Center for Cancer/Hebei Cancer Hospital, Chinese Academy of Medical Sciences, Langfang, 065001 China; 3 New Research Building, National Key Laboratory of Molecular Oncology, National Cancer Center/National Clinical Research Center for Cancer/Cancer Hospital, Chinese Academy of Medical Sciences and Peking Union Medical College, 17 Panjiayuan Nanli, Chaoyang District, Beijing, 100021 China

**Keywords:** Colorectal cancer, Liver metastasis, Genetic alteration, Cancer stem cell, Microenvironment, Biomarkers

## Abstract

Metastasis is the leading cause of death in colorectal cancer (CRC) patients, and the liver is the most common site of metastasis. Tumor cell metastasis can be thought of as an invasion-metastasis cascade and metastatic organotropism is thought to be a process that relies on the intrinsic properties of tumor cells and their interactions with molecules and cells in the microenvironment. Many studies have provided new insights into the molecular mechanism and contributing factors involved in CRC liver metastasis for a better understanding of the organ-specific metastasis process. The purpose of this review is to summarize the theories that explain CRC liver metastasis at multiple molecular dimensions (including genetic and non-genetic factors), as well as the main factors that cause CRC liver metastasis. Many findings suggest that metastasis may occur earlier than expected and with specific organ-anchoring property. The emergence of potential metastatic clones, the timing of dissemination, and the distinct routes of metastasis have been explained by genomic studies. The main force of CRC liver metastasis is also thought to be epigenetic alterations and dynamic phenotypic traits. Furthermore, we review key extrinsic factors that influence CRC cell metastasis and liver tropisms, such as pre-niches, tumor stromal cells, adhesion molecules, and immune/inflammatory responses in the tumor microenvironment. In addition, biomarkers associated with early diagnosis, prognosis, and recurrence of liver metastasis from CRC are summarized to enlighten potential clinical practice, including some markers that can be used as therapeutic targets to provide new perspectives for the treatment strategies of CRC liver metastasis.

## Background

Colorectal cancer (CRC) is the world’s second leading cause of cancer deaths, and liver metastasis from CRC accounts for the majority of fatalities in CRC patients [1, 2]. CRC’s most common target metastatic sites are the liver, lung, bone, and brain, known as organ tropism, and the liver is the most common site of CRC metastasis [3]. Up to 50% of CRC patients have liver metastasis, and approximately 15–23% of patients have metastasis at the time of diagnosis. Hepatic resection combined with modern adjuvant systemic regimens is only effective in 20% of colorectal cancer liver metastasis (CRLM) patients. Even after curative hepatic resection, the 5-year overall survival rate is around 48% [4]. In practice, however, approximately 80% of CRLM patients have unresectable metastatic lesions [5, 6].They are typically downstaged by systemic and local therapy (including stereotactic radiotherapy, transarterial radioembolization, and transarterial chemoembolization) to achieve metastatic liver lesion resection, improving patients’ long-term survival and prognosis. Although the development of adjuvant systemic therapy has significantly improved the clinical outcome of patients with stage IV CRC liver metastases [7], early detection of CRLM remains critical to good results. Despite the high resolution of computed tomography (CT) as the most commonly used detection modality, up to 30% of liver metastasis cannot be detected in their early stages. MRI and PET-CT may have higher sensitivity and specificity, but the costs are prohibitive [2].

Many studies have found that cancer metastasis is a complex selective process influenced by anatomical, biological, and microenvironmental factors [8]. Theories such as “seed-soil,” pre-niche, and the crosstalk between tumor cells and immune cells provide direct evidence for metastatic propensity and organ-specific tropism of metastatic cancer cells, implying that metastasis is a process that is dependent on organ-targeted anchoring characteristics [9–11]. Thus, investigating how metastatic CRC appears, when it appears, and the underlying mechanism provides clues for the treatment of CRC and its liver metastasis.

## Theories underlying organ-specific metastasis process

**Fig. 1:**
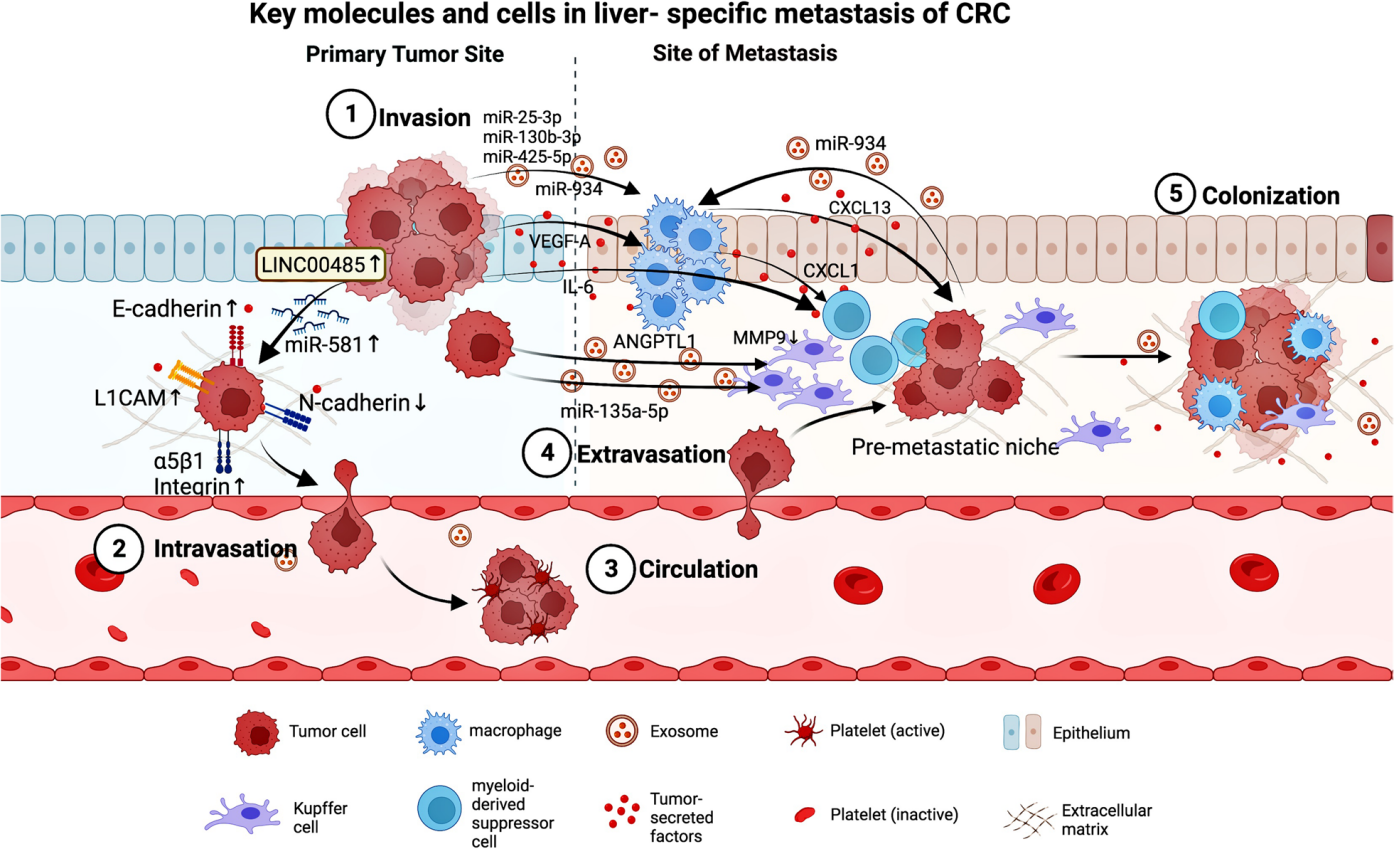
Key molecules and cells for liver-specific metastasis of CRC. Invasive cancer cells from primary CRC tumors invade the adjacent vasculature from the primary site. The invading tumor cells circulate within the blood vessels protected by platelets, extravasate, and finally colonize the liver. The key molecules and cells involved in liver-specific metastasis of CRC include immune cells (MDSCs, TAMs, Kupffer cells), cytokines (IL-6, VEGF-A), chemokines (CXCL13, CXCL1, CXCL12), exosomes (miR-25-3p, miR-130b-3p, miR-425-5p, miR-934, miR-135a-5p, miR581, ANGPTL1) and cell adhesion molecules (L1CAM, E-cadherin, α5β1 Integrin and N-cadherin). Several molecular and cellular interactions have been identified to play critical roles in CRC liver metastasis, and these factors may influence the organ tropism of CRC metastasis

According to a traditional model of metastatic spread, cancer cells undergo the following general steps known as metastatic cascade, which can be divided into two major phases: (1) dissemination from the primary lesion to distant organs by entering systemic circulation and adapting to a new microenvironment (intravasation, extravasation), and (2) colonization followed by expanding growth [12]. The multi-step process typically involves an invasion-metastasis cascade. According to Hanahan and Weinberg, the hallmark of “activating invasion and metastasis” is one of the six important features of cancer. Invading cancer cells pass through or collaborate with stroma to avoid elimination by immune system cells such as neutrophils, monocytes/macrophages, and endothelial cells. Epithelial-mesenchymal transition (EMT) program can be activated by carcinoma cells and orchestrate most steps of invasion and metastasis, except for colonization. Disseminated tumor cells act in dormancy in circulation and new environment tissue to avoid immune surveillance, and then they interact with the tissue microenvironment to be awakened from dormancy. Moreover, the development of metastatic colonization needs multiple biological programs, and these adaptations require intrinsic capabilities of cancer cells and a permissive tumor microenvironment with stromal support cells. The invasion-metastasis process, which appears to be a linear progression from the primary tumor to metastatic colonies, operates under the guidance of a specific paradigm [13–15]. However, several studies have suggested that metastasis cannot be defined solely by chronological order, as many interdigitated and mutually exclusive metastasis events do not appear to follow a linear progression model [16]. In our understanding of CRC metastasis to the liver, the general “seed and soil” theory and the “mechanistic theory” are highly complementary [17]. Anatomically, CRC metastasis is thought to occur in a stepwise fashion, with the majority of venous drainage from the intestine entering the portal system to flow into the liver, and then the disseminated cells in the bloodstream are arrested by the first available liver capillary beds with endothelial cells and basement membrane [18]. Although the physical characteristics influence organ tropism and affect the non-random organ-specificity, the fact is that organs receiving similar blood volumes have distinct metastatic-formation efficacy. The special ability of circulating tumor cells to form secondary growth cannot be explained as purely mechanistic [19, 20]. Cancer cells entering the circulation disperse in various directions, but their anchorage to specific metastatic sites is determined by various factors [21]. Stephen Paget proposed a hypothesis in 1889 that described cancer cells as “seeds” and receptive microenvironments as “soils,” both of which are required for rate-limiting steps in the formation of micrometastasis [9]. Recently, growing evidence for the process of pre-metastatic niche formation adds new insights to the “seed-soil” theory. For example, metastasis-initiating cells co-opt the metastatic microenvironment to facilitate colonization. Before cancer cells disseminate from the primary tumor, a subpopulation of primary tumor cells has the “prime” potential and reprograms the distant microenvironments [22]. Pre-metastatic niche formation is important in CRLM. By recruiting various cellular components (Kupffer cells, macrophages, and fibroblasts), producing CRC-derived factors (chemokines and cytokines) and exosomes, primary CRC prepares a favorable microenvironment in the liver. It mediates liver-target metastasis [23, 24]. As a result, the concept of pre-metastasis niches may trump the chronological metastasis pattern that is dependent on circulation. Recently, many discoveries have clarified the key molecules and cells involved in liver-specific metastasis of CRC. For example, LINC00485 is a newly discovered class of Long non-coding RNAs (lncRNAs), and low expression of LINC00485 predicts a poor prognosis for CRC patients. LINC00485 attenuated CRC cell invasion and liver metastasis by directly modulating the miR-581/EDEM1 axis. Overexpression of LINC00485 enhanced the expression of epithelium markers E-cadherin and significantly down-regulated the expression of mesenchymal markers N-cadherin, indicating a loss of malignant phenotype in cancer cells [25]. Higher levels of tumor suppressor microRNAs (miR-25-3p, miR-130b-3p, miR-425-5p, miR-934) in the exosomes were secreted by CRC cells in more advanced disease, and these exosomal miRNAs induced macrophages M2 polarization to promote liver metastasis of CRC. CXCL13 secreted by M2-polarized macrophages promoted the transcription of exosomal miR-934 in CRC cells, forming a positive feedback loop to foster CRLM [26, 27]. Recent studies have revealed the complex interactions between immune cells (myeloid-derived suppressor cells, macrophages, Kupffer cells) and cancer cells in the tumor microenvironment. For example, primary CRC tumor-secreted VEGF-A stimulated tumor-associated macrophages to produce CXCL1, which recruited CXCR2-positive myeloid-derived suppressor cells (MDSCs) accumulated in the pre-metastatic site and facilitated liver metastasis [28]. Furthermore, a high level of IL-6 secreted by tumor cells recruited MDSCs to accumulate in the pre-metastatic niche in the liver, which was stimulated by the S1PR1–STAT3 signaling pathway and positively correlated with the number of metastatic liver nodes. Increased CD14^+^HLA-DR^−/low^ MDSCs in CRLM patients were shown to inhibit T-cell proliferation and predict poor prognosis [10]. ANGPTL1 was significantly down-regulated in CRC-derived exosomes. It attenuated CRLM by reprogramming Kupffer cells (KCs) to reduce their MMP9 expression, which helped prevent vascular leakage [29]. Under hypoxic conditions, exosomal miR-135a-5p was released from primary tumor cells and phagocytosed by KCs, which selectively initiated a favorable pre-metastatic formation in the liver by establishing an immunosuppressive microenvironment [30]. In addition, some cell adhesion molecules have been demonstrated to play an important role in CRC liver metastasis. It has been reported that α5β1 Integrin was highly expressed in metastatic CRC cells [31]. E-cadherin ensures the epithelial integrity, and membrane E-cadherin in metastatic cancer cells is lost during tissue dissociation or tumor invasion. Disruption of E-cadherin relieved REST-mediated repression of L1CAM and upregulated L1CAM expression [32] (Fig. 1).

Metastasis is recognized as an evolutionary process, with the genomes carrying the archeological record of each metastatic cancer cell. Indeed, many genomic studies have been conducted to explain metastatic subclones’ emergence and and investigate the “timing, route, and direction” of metastatic spread [33]. It appears that the metastasis process is not constrained to a specific model of evolution but rather follows complex evolution patterns, which means that single or multiple clone(s) with metastatic traits leave the primary tumor at distinct timing points and form metastases in distant organs via various trajectories, including monophyletic seeding and polyphyletic seeding [34, 35]. The relative timing of metastatic spread is determined by comparing the genetic divergence between the primary tumors and metastasis. In the classic “linear evolution model,” the metastasis-initiating clone(s) emerge late in the primary tumor and seed at the metastatic sites as a byproduct of tumor development. Instead, in the “parallel evolution model,” the metastatic subclone(s) spread from the primary tumor to distant sites early, and both the primary and metastatic subclones evolve concurrently. As a result, compared with the “linear evolution model,” a greater degree of Primary-Metastasis genetic divergence is expected in the “parallel evolution model” [36]. In addition to the time of metastasis, studies of the clonal relationship between primary tumors and metastases explained metastatic seeding patterns based on genomic data analysis: identification of monoclonal/polyclonal metastasis and monophyletic/polyphyletic metastasis may provide information for treatment improvement. In metastatic CRC, both monoclonal/polyclonal metastasis and monophyletic/polyphyletic seeding patterns were observed. Polyclonal metastasis appeared to be the most common type of CRC metastasis. A polyphyletic seeding pattern was observed in the case of CRC with liver metastasis followed by lung metastasis [37, 38]. For the distinct routes of metastasis, Hai-ning et al. investigated the genomic evolution for the clonal origin and revealed three metastatic models (sequential, branch-off, and diaspora) by phylogenetic reconstruction using Treeomics. The results of the genomic analysis showed that liver and lung metastasis might originate from primary tumors independently rather than subsequently, providing genomic evidence for the organotropisms of metastatic CRC cells. However, the relationship between the characteristics of primary site subclones and their potential for liver metastasis has not been thoroughly investigated [38].

## The underlying molecular mechanisms and contributing factors involved in CRLM

### Genetic and epigenetic changes associated with liver metastasis of CRC

Cancer cells are thought to acquire metastatic capacity due to genetic and epigenetic changes. Genetic mutations can potentially disrupt epigenetic patterns, and the interaction between these two mechanisms can promote metastasis. [33, 39–42].

Although numerous genetic alterations have been detected between the primary tumor and metastatic sites in CRC [43–45], much remains unknown about the interaction between tumor genomic features and metastatic potential and organ-specific metastatic patterns [34, 46]. The clonal relationship observed between paired primary tumors, and metastasis explains at least part of the dissemination of metastasis-competent clones in different temporal patterns and trajectories [36, 47, 48]. This section will will sort out the genetic/epigenetic alterations associated with CRC liver-specific metastasis and summarize recent studies on the metastatic evolution patterns (temporal patterns and routes) observed in the metastatic CRC cohort.

### Genetic alterations associated with liver metastasis of CRC

Several whole-genome sequencing analyses on metastatic tumors have been performed in recent years to gain insight into the critical genetic events involved in CRLM. Several studies have been conducted to investigate single nucleotide variations (SNVs), mutated genes, and chromosome copy numbers of CRLM. An analysis of metastatic solid tumor genomes revealed that consistent genetic changes indicate cancer metastasis remains to be further identified [44]. A pan-cancer cohort study of 25,000 patients’ tumor genomic profiling identified the associations between genetic alterations and metastatic patterns in 50 tumor types. The result showed that copy number alterations were not significantly associated with the metastatic burden for CRC. Chromosomal instability may be established early in tumor development and was already high in patients with low metastatic burden [45]. Oga et al. discovered 6855 mutations in primary CRC tumors without liver metastasis, primary metastatic CRC, and paired liver metastasis (LM) lesions using whole-exome sequencing (WES) analysis. The result showed that the somatic genomic profiles of primary CRC tumors and LM lesions were not significantly different; however, LM regions showed an enriched A-to-C nucleotide conversion in the context of “AAG,” an event that may be specific to liver metastases [49]. Li et al. used WES to look for somatic SNVs (sSNVs) in primary tumors and matched liver metastasis samples from 16 CRC patients with liver metastasis. The SNVs data were analyzed using ABSOLUTE software to calculate the proportion of mutational genes in each sample. An average of 34% (8–63%) mutations were shared by both primary tumors and liver metastasis, indicating a common ancestral trunk among them. Furthermore, an average of 34% (12%–88%) mutations were metastasis-private, which may be a result obtained or lost during the tumor metastasis process. The probable timing order of mutation events has been investigated by analyzing the distribution of cancer cell fractions (CCF). A higher median CCF value indicates that the mutation occurred earlier. Data on the median CCF value of TP53 and KRAS showed that TP53 mutations occurred earlier than KRAS in primary tumors but later than KRAS in liver metastasis [50].

Several studies have been conducted to identify frequently mutated genes involved in metastasis. For example, 707 genes have been identified as LM-associated genes, which specifically mutated in the LM regions but not in CRC tumors without liver metastasis, including ADAMTS10, NELL1, and RXFP3, implying their roles in liver metastasis. Furthermore, ADAP1 fusions were discovered in the RNA-seq dataset, indicating that ADAP1 was fused to GET4, SUN1, or NOC4L in an out-of-frame manner in the LM region. Two in-frame fusions of the ADAP1’s ArfGAP domain with proteins from GEMIN4 and TMEM8A have been discovered, which may facilitate metastasis by activating GTPase [49]. A study used targeted sequencing of primary tumors and matched liver metastasis samples to describe the genome landscape of Chinese CRLM patients. The most frequently mutated genes were found to be TP53 (324/396, 82%), PC (302/396, 76%), KRAS (166/396, 42%), SMAD4 (54/396, 14%), FLG (52/396, 13%), and FBXW7 (43/396, 11%). Furthermore, the distribution of genomic changes was related to the time of metastasis (synchronous/metachronous liver metastasis). Alterations in genes of FBXW7, FLT3, XIRP2, TSC2, LATS1, and CREBBP were significantly enriched in metachronous lesions, and alterations in CDK12 were significantly enriched in synchronous LM [51].

The differences in chromosome copy number between primary and secondary tumors revealed that genetic aberrations in liver metastasis are a dynamic process, such as the presence of a focal amplification of chromosome 7p in primary tumors but not in the LM region. The loss or gain of copy number variations (CNVs) most likely allows clones to be more fit in a new environment [49]. Anand and colleagues investigated the link between aneuploidy and CRC metastasis. Aneuploidy is not just a byproduct of chromosomal instability; it has a direct influence on cancer cells’ metastatic capability, either promoting or inhibiting metastasis behavior. HCT116 colon cells with an extra copy of chromosome 5 exhibit increased invasive behavior by activating an EMT program and upregulated matrix metalloproteinases (MMPs) [52]. In addition, CNV alterations, as a common biological event during tumor progression and therapy, usually involve multiple genes. There are potentially complex interactions between co-amplified or co-deleted genes affected by CNV events, acting as a whole. It has been reported that CDK12 and HER2 were frequently co-amplified in CRC, and inhibition of CDK12 can enhance the sensitivity of CRC cells to lapatinib, an anti-HER2 tyrosine kinase inhibitor (TKI) [53].

### Genome events related to metastatic evolution pattern

According to phylogenetic analysis of non-synonymous SNVs from the primary tumor and metastatic liver lesions, there were three main clonal evolution patterns from primary to liver metastases: clonal-clonal pattern (C–C) (early events), subclonal-clonal pattern (S–C) (middle-stage events), none-clonal pattern (0-C) (later events). In terms of CNV events, Chr 20q amp, 17p del, 18q del, and 8p del in clonal- clonal evolution were considered as early events, 8q amp in liver metastasis-specific evolution was considered as later events, and 8q amp, 13q amp, and 8p del in subclonal-clonal evolution were considered as middle-stage events. SYNE1 was a mutant gene with S–C clonal evolution characteristics. Its mRNA expression level in normal, CRC primary, and liver metastasis gradually decreased; however, its functional mechanism in CRLM remains unknown [50]. Tumor mutation burden, an immunotherapy biomarker, in conjunction with HLALOH (HLA, Human leukocytes antigens, LOH, Loss of heterozygosity), is used as an indicator to assess the efficacy of immunotherapy [54]. Subclonal mutation loads were higher in primary tumors than in clonal mutation loads. In contrast, the proportion of clonal mutation was increased in metastatic lesions, which is consistent with the S–C evolutionary pattern, indicating the role of selection in metastasis. HLA LOH occurred in samples with recurrent mutations of S–C changing pattern, including KRAS, SYNE1, FBXL2, DNAH11, and CACNA1H, indicating that this mutational clonal pattern promotes CRC cells evading the immune system during liver metastasis [50].

### Epigenetic modifications associated with liver metastasis of CRC

**Fig. 2:**
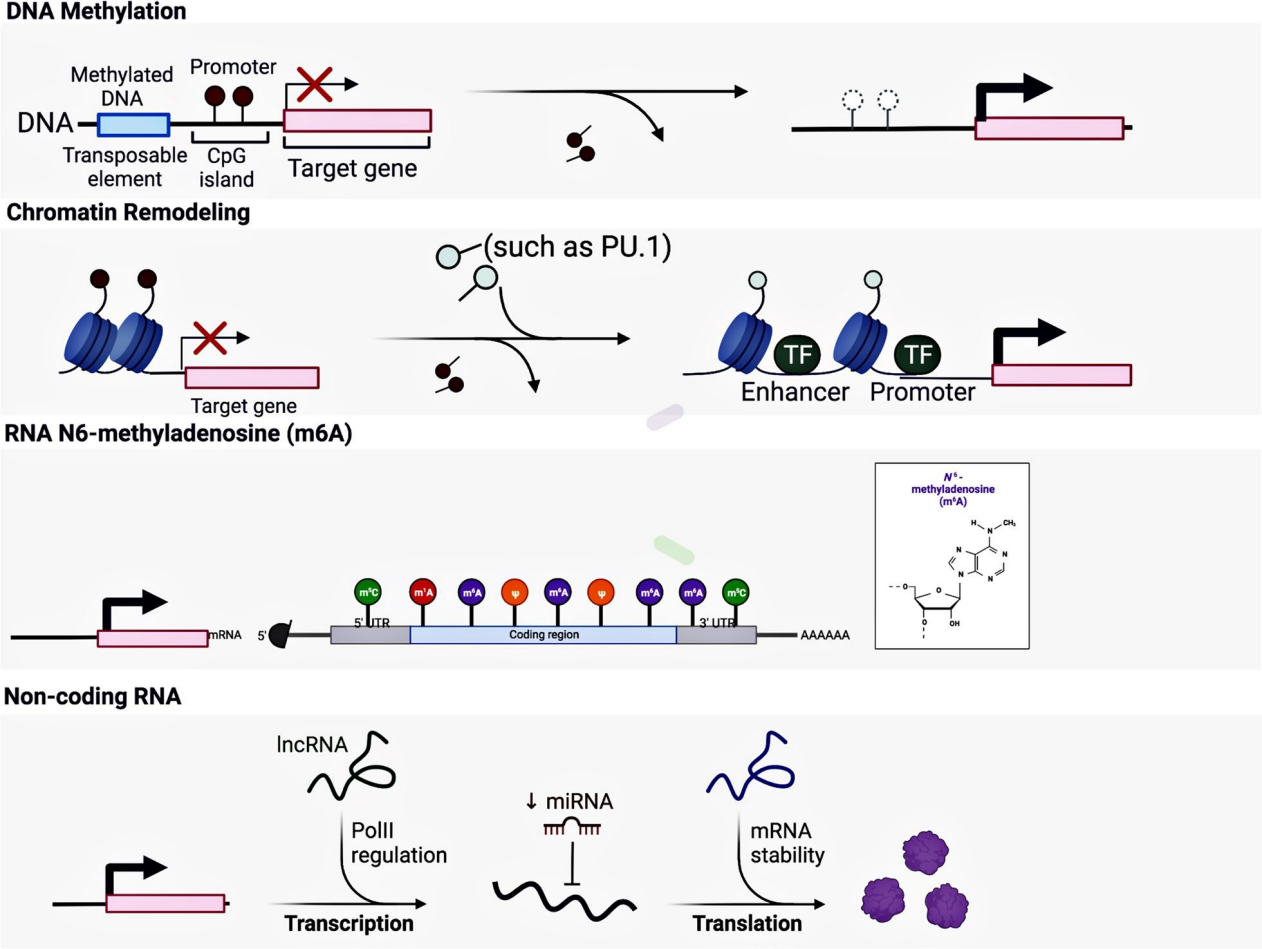
The role of epigenetic modifications in CRC liver metastasis. Epigenetic modification plays a vital role in gene regulation, mainly for various covalent modifications of histones and nucleic acids. The change of nucleic acid is in DNA and RNA. In addition, epigenetic modification also includes chromatin remodeling, non-coding RNA regulation, and other mechanisms. DNA methylation mainly occurs at the C of 5′-CpG-3′ to generate 5-methylcytosine (5mC). Under the action of DNA methyltransferase (DNMT), methyl groups are covalently bonded to the 5' carbon of cytosines of CpG dinucleotide residues. Hypermethylated gene expression is suppressed. Chromatin remodeling can regulate gene expression by regulating chromatin changes in chromatin structure and location, such as PU.1 opening chromatin regions of downstream effector genes and recruiting additional epigenetic modifiers to regulate gene expression. N6-Adenylate methylation (m^6^A), which inserts a methyl substituent on the N atom at the 6-position of adenosine. During transcription, m^6^A deposited on RNA transcripts affects gene expression post-transcriptionally by altering the structure of RNA or the specific recognition of m6-binding proteins. Non-coding RNAs are endogenous RNA molecules that cannot be translated into proteins but have particular gene expression regulatory functions, regulating post-transcriptional gene expression by complementary binding to RNA transcripts of the target gene

No genetic changes have been identified as consensus metastasis-specific drivers in the process of CRC metastasis. However, epigenetic changes may provide an alternative mechanism to induce tumor cells for metastatic phenotypes [55]. The core content of epigenetic modification is the covalent modification of histones and nucleic acids (including methylation, acetylation, ubiquitination, etc.). In addition, epigenetic regulation also includes chromatin remodeling and transcriptional mediators (mainly non-coding RNAs, such as microRNAs and long ncRNAs) of the RNA splicing machinery. They affect gene expression without sequence changes in DNA [56, 57] (Fig. 2). Epigenetic changes play an important role in CRLM (Table 1), but whether there are metastasis-specific epigenetic drivers and their mechanism need to be investigated further [40].

**Table 1:** Epigenetic modification in CRC liver metastasis

	Upstream regulator	Targets	Biological function	Refs.
Chromatin remodeling	PU.1	DPP4	PU.1 promotes DPP4 expression by increasing histone acetylation at the DPP4 locus, which in turn promotes CRC liver metastasis	[58]
m^6^A modification	YTHDF1	ARH	YTHDF1 enhances ARHGEF2 translation by binding to the m^6^A site of ARHGEF2 messenger RNA, promoting CRC tumorigenesis and metastasis	[59]
	circNSUN2	HMGA2 mRNA	circNSUN2 enhances the stability of HMGA2 mRNA to promote CRC metastasis progression	[60]
ncRNA modification	miR-146a	c-met	miR-146a directly targets c-met to reduce CRC tumor cell invasion and inhibit CRC liver metastasis	[61]
	circPPP1R12A-73aa	MST1/2, LATS1/2	circPPP1R12A-73aa inhibits MST1/2 and LATS1/2 activities and activates the Hippo-YAP signaling pathway to promote CRC metastasis	[62]
	LncRNA- CYTOR	β-catenin	LncRNA-CYTOR interacts with β-catenin to activate Wnt/β-catenin signaling, promoting epithelial-mesenchymal transition (EMT) and metastasis in CRC	[63]

Dysregulation in DNA methylation is the mainly studied DNA modification in tumor and metastasis [64]. The methylation changes of primary CRC, metastatic CRC, and liver metastases differ between individuals. CRC primary tumors exhibited global hypomethylation and CpG island (CGI) hypermethylation compared to healthy tissues, whereas metastatic colorectal lesions exhibit high-level global methylation but lower CGI methylation [65]. The study by Udali et al. came to the same conclusion. Primary CRC and synchronous liver metastases had similar epigenetic DNA hypomethylation status when compared with homologous cancer-free colon tissues, indicating that these epigenetic mechanisms occurred in the early stages of CRC development and were maintained till the stage of liver metastasis progression [66]. However, the mechanism by which methylation inhibits tumor suppressor gene expression may be compromised during metastasis. Mahdi et al. discovered that the regulatory mechanism of methylation on gene expression might be compromised during the process of CRC tumor cell metastasis and colonization in the liver. The expression levels of three endothelin system genes changed significantly during the liver colonization of CC531 cells. When metastatic cell lines were exposed to Decitabine (DAC, which inhibits DNA methyltransferases), the expression of endothelin system genes did not increase, indicating that these gene expression changes were not caused by DNA methylation. This suggests that the regulatory function of epigenetic alterations may be gradually lost in the late stage of metastasis [67]. The microenvironment-induced epigenetic mutation is an essential mechanism for metastatic tumor cells to grow in their new niche. The hepatic growth factor (HGF) is abundant in the microenvironment of liver metastases. HGF from the metastatic liver microenvironment was shown to activate the c-Met/PI3K/AKT/mTOR axis in CRC cells, activating the SREBP2-dependent cholesterol biosynthesis pathway to promote CRC liver metastasis [68]. PU.1 is a pioneer factor that remodels chromosomes by opening the enclosed chromatin and enlisting the help of additional epigenetic modifiers. According to one study, HGF caused PU.1 phosphorylation in metastatic cells. The phosphorylated PU.1 regulated downstream regulatory elements to activate the effector gene DPP4. The HGF/PU.1/DPP4 axis was activated, which promoted the growth of CRC tumor cells at the site of the liver. Targeting the chromatin remodeling pathway in the future may provide additional treatment options for metastatic cancer [58].

RNA N6-methyladenosine (m^6^A) is the most prevalent internal modification in messenger (m)RNA in eukaryotes, and recent studies have shown that m^6^A modification also exists in lncRNAs, Circular RNAs (circRNAs), and pre- Micro RNAs (pre-miRNAs), and plays essential roles for their biogenesis and functions [69]. In CRC, m^6^A modification accelerates CRC progression and metastasis by promoting glycolysis in cancer cells, inducing immunosuppression in the tumor microenvironment, maintaining tumor cell stemness, and promoting chemoresistance [64]. One study found that YTHDF1, a translation-promoting cytoplasmic m^6^A reader, was the most significantly upregulated m^6^A regulator in CRC cells. YTHDF1 activated RhoA signaling by binding to the m^6^A site of ARHGEF2 messenger RNA, leading to enhanced translation of ARHGEF2, and the YTHDF1-m^6^A-ARHGEF2 axis promoted the growth of CRC cell lines in primary organs as well as in lung and liver metastases [59]. circNSUN2 was found to be frequently upregulated in tumor tissue and serum samples from CRC patients with liver metastases, suggesting poor overall survival. The experimental results showed that the N6-methyladenosine modification of circNSUN2 regulated its cytoplasmic export and enhanced the stability of HMGA2 mRNA by forming a circNSUN2/IGF2BP2/HMGA2 RNA–protein ternary complex, which led to the LM of CRC. And circNSUN2 may serve as a novel therapeutic target of CRLM, providing treatment options for patients with CRLM [60].

The role of miRNA expression and miRNA-gene regulation in the progression and metastasis of CRC is relatively well understood. Anne-Marie et al. demonstrated that c-met was a direct target of miR-146a. Overexpression of miR-146a inhibited the expression of the proto-oncogene c-met, which reduced the development of liver metastasis and may be used as a therapeutic target for treating CRLM [61]. Lee et al. compared the miRNA and gene expression profiles of two groups of primary tumor samples with and without metastasis to identify the miRNA-target regulators to initiate metastasis [70]. The miR-424 has previously been shown to be upregulated and specifically target FGFR1 to inhibit its expression in placental trophoblasts [71], which is consistent with the miRNA-target network results. While PDGFRB inhibited miR-30b expression, which may be linked to the initiation of CRLM, miR-30b has been shown to promote cancer cell apoptosis in human gastric cancer tissues [72]. Circ RNAs have been shown to contain many miRNA binding sites and function as miRNA sponges, allowing them to indirectly regulate miRNA-target genes' expression. It has also been demonstrated that it encodes peptides with regulatory procedures. Zheng et al. discovered that the expression of circPPP1R12A, which encoded a conserved 73-aa small peptide, circPPP1R12A-73aa, was significantly increased in primary colon cancer tissues. Not circRNA circPPP1R12A itself but circPPP1R12A-73aa can promote CRC metastasis by inhibiting MST1/2 and LATS1/2 to activate the Hippo-YAP signaling pathway, which may be used as a therapeutic target for CRLM [62]. LncRNAs modulate the biological process of tumor metastasis via mechanisms such as transcriptional regulation and chromosome modification. Ben Yue and colleagues discovered a new positive feed-forward loop mechanism between LncRNA cytoskeleton regulator RNA (CYTOR) and Wnt/β-catenin signaling that promotes EMT phenotype and CRC metastasis. Targeting the CYTOR/β-catenin axis may be a promising treatment strategy for CRLM [63].

### Dynamic stem cell hierarchies and stem cell states in CRLM

Several cellular properties, including cancer stem cell (CSC)-like traits, EMT, and autophagy, among others, are linked to the acquisition of metastatic ability [73, 74]. According to the cancer stem cells hypothesis, CSC-like properties (self-renewal ability and cellular plasticity) of primary tumor cells are an obvious prerequisite for tumor initiation and metastatic clone formation [75, 76].

#### Dynamic hierarchical and phenotypic changes related to cell plasticity in liver metastasis of CRC

**Fig. 3:**
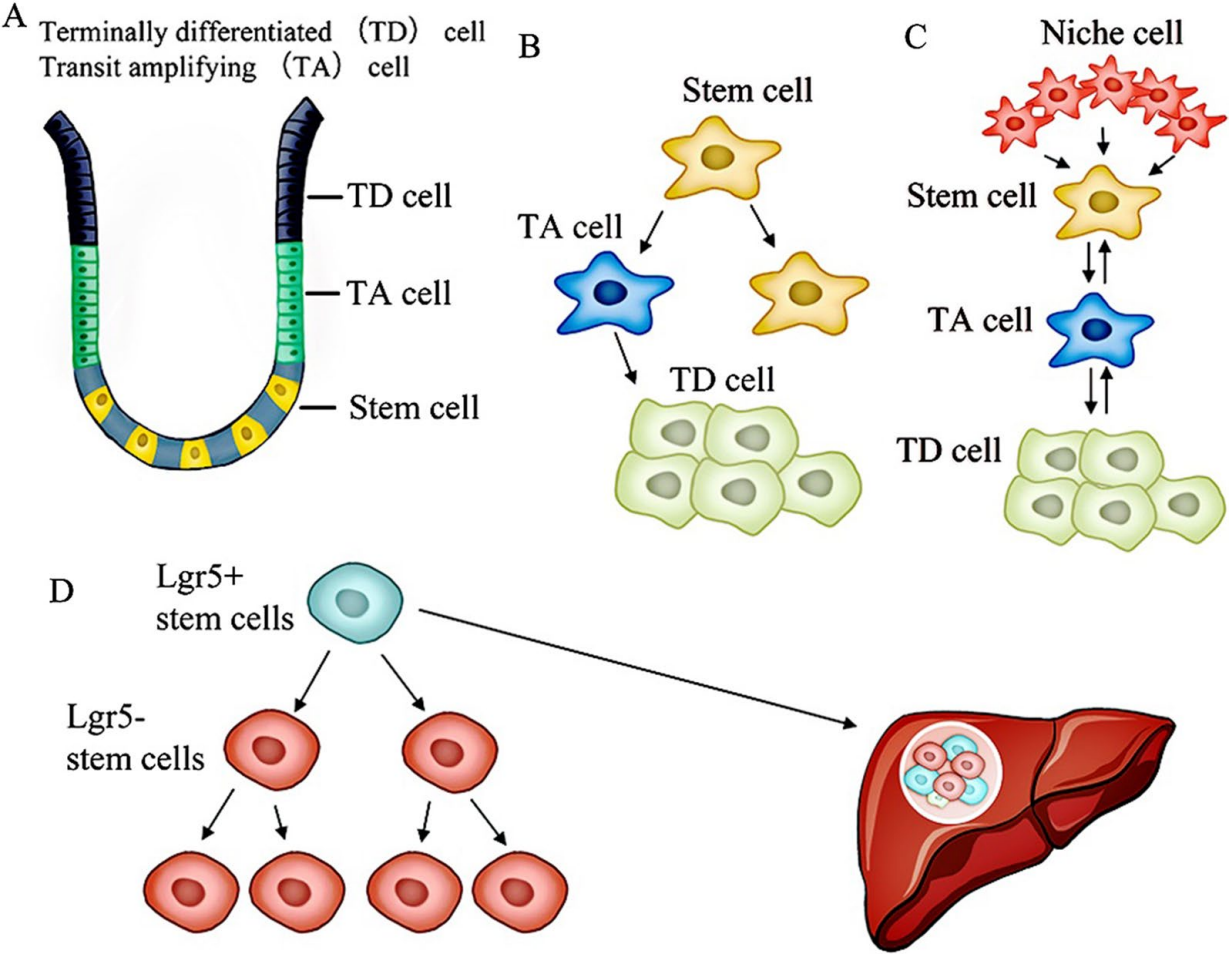
Dynamic hierarchy and phenotypic plasticity of CSCs are prerequisites for CRLM. The intestinal crypt-villus structure is a dynamic stem cell hierarchical organization essential in colorectal cancer progression and metastasis. The compartment of hierarchical organization of colorectal cancer contains tumor cells in different states: SC cells, TA cells, and TD cells. Stem cells (SC) at the apex of hierarchies generate transient amplifying (TA) progenitor cells, and TA cells differentiate into terminally differentiated (TD) cells. B and C represent the hardwired stem cell hierarchy and the novel dynamic stem cell hierarchy, respectively. The latter model has a more plastic stem cell hierarchy, meaning that TA and TD cells can dedifferentiate to replace lost SCs through reprogramming. D. The role of cellular plasticity in colorectal cancer metastasis. CRC models demonstrate that Lgr5 + CSCs initiate and maintain metastasis, but tumor cells that escape the primary tumor and disseminated cells found in the blood circulation are overwhelmingly Lgr5- cells. Lgr5- cells were seeded in the metastatic site, and Lgr5 + cells appeared in the metastatic site after development period. When Lgr5 + cells are lost, Lgr5- cells replenish the eliminated Lgr5 + cells by dedifferentiation

The dominant view of hierarchical organization is a critical concept for understanding the role of cellular plasticity within cancer stem cells, differentiated cells, and cells in the intermediate state, which reflects cell–cell cooperation between different clones [77]. Many studies have shown that both primary tumors and metastatic lesions of CRC have hierarchically organized structures with heterogeneous cell populations (CSCs, niche cells, transient amplifying cells, and differentiated cells) in dedicated niches (Fig. 3A). According to the “hardwired stem cell hierarchy” model, it believes that CSCs are located at the bottom of the structure (intestinal soil crypts), split asymmetrically into a CSC and a transient amplification (TA) cell, with the latter dedicated to differentiation (Fig. 3B). In contrast, increasing evidence supports the “CSC-driven tumor hierarchy” model, which allows daughter cells (TA cells) and fully differentiated cells to dedifferentiate into stem cells to replenish the damaged CSC pool [78–80] (Fig. 3C). Many studies have shown that CSCs are the root of CRC metastasis. For example, according to Dieter et al. research, three types of tumor-initiating cells (TICs) with stem-like properties were identified, and only the long-term TICs (LT-TICs) characterized as extensively self-renewing potential was assumed to drive CRC metastasis [76].

#### Dynamic phenotypic properties of stem cells

Many recent studies have looked into the relationships between CSCs and non-CSCs, as well as the role of cellular plasticity in CRC with metastasis. CSCs, as the tumor’s clonogenic core, have specific markers such as leucine-rich repeat-containing G-protein-coupled receptor 5 (LGR5). Because selective depletion of colon Lgr5 + CSCs prevented liver metastasis, the experimental evidence has proven that colon cancer-derived liver metastasis depends on a specific subpopulation of Lgr5 + cells. However, once the Lgr5 + cell consumption was stopped, the tumor regenerated rapidly, indicating that the tumor cannot be eliminated by ablating Lgr5 + cells. A study further discovered that colorectal tumors were maintained by proliferative Lgr5 − cells. Lgr5 − cells replenished the Lgr5 + cell pools to promote tumor growth. Furthermore, other experiments revealed a dynamic phenotype of Lgr5 cells that showed that Lgr5 re-expressed after arriving the support microenvironmrnt of the stem cell niche, but the most disseminated and seeding cells are Lgr5 − rather than Lgr5 + . Finally, both Lgr5 − and Lgr5 + cells were observed enriched in liver metastasis, and Lgr5 + cells were required for metastatic tumor growth, indicating that cellular plasticity is a deterministic step in the metastasis process [81] (Fig. 3D).

The phenotypic properties of stem cells (such as CD44) [82] and their interaction with the microenvironment remain unknown. Fixed CSCs and migrating CSCs (MCSCs) are the two supposed types of CSCs. Gao et al. identified organ-specific metastatic MCSCs in human CRC. CD110 + cells have stem cell characteristics that promote liver metastasis, which may be used as a surface marker of liver-specific metastasis [83]. Intestinal stem cells (ISC) located at the bottom of the mucosal invagination maintain intestinal tissue regeneration. EphB2 receptors were highly expressed in ISC, and EphB2 + ISC-like tumor cells promoted CRC recurrence after primary tumor excision in immunocompromised mice [84]. Compared with normal tissues, HIST2H2BF protein was overexpressed in CRC tissues and was associated with a poor prognosis in the patients. HIST2H2BF may promote the development of stem cell-like properties by activating the Notch pathway, indicating its therapeutic potential [85]. Similarly, Wang et al. discovered BMI-1 expressed on the surface of cancer cells in primary colorectal tumors and played an essential role in the self-renewal of CRC stem cells. And because inhibiting BMI-1 with the small molecule compound QW24 can reduce CRC metastasis to the liver, BMI-1 may be used as a new therapeutic target for CRLM [86].

### Microenvironment factors associated with CRC liver metastasis: pre-niche, CAFs, and inflammation/immune response

The tumor microenvironment includes innate and adaptive immune cells, stromal cells, endothelial cells, and cancer-associated fibroblasts. Since crosstalk between tumor cells and immune cells ultimately results in an environment that promotes tumor growth and metastasis, the tumor microenvironment is closely related to tumor formation and metastasis [87]. CRLM is frequently regarded as a late-stage random event. And it is believed that metastatic clones emerge late and are formed by the long-term evolution of primary tumor cells. Then the disseminated cancer cells circulate through the portal vein into the liver [88]. However, new evidence suggested that tumor metastasis may occur earlier than expected, which is influenced by both the intrinsic factors of tumor cells and the tumor microenvironment, such as pre-niche formation [10], whose related molecular and cellular events also help to explain the organotropism of CRC metastasis [24, 89]. The appearance of invisible micrometastases (1–2 mm) is caused by disseminated tumor cells choosing to enter a dormant period in the new microenvironment, influenced by molecules and cells. FBX8 is a member of the F-box protein family and directly binds to proteins such as HIF-1, CDK4, and C-myc via its Sec7 domain, promoting the dormancy of liver metastatic CRC cells [90]. Targeting dormant cells to inhibit colonization provides a new theory for treating CRLM.

#### Pre-niche formation in liver metastases

Seed-soil theory and pre-niche theory both support the idea that cancer metastasis is not random but rather organ-specific [91]. Some researchers have found that primary CRC tumors can promote the formation of hepatic pre-niches by releasing miRNA-containing exosomes [92]. Furthermore, the primary tumor’s pro-inflammatory cytokines, chemoattractants, and angiogenic factors are thought to initiate the formation of the pre-niche [93], recruiting and mobilizing bone marrow-derived cells, and marrow-derived granulocytic MDSCs to the target metastasis site. MDSCs have been demonstrated to contribute to inflammation-associated tumorigenesis and metastasis through multiple mechanisms [94]; Wang et al. discovered that primary CRC tumors secrete VEGF-A, which stimulated macrophages to secrete CXCL1. CXCR2 is the CXCL1 receptor found on the surface of MDSC. CXCL1 overexpression attracted MDSC to the metastatic liver site. Targeting CXCR2 on MDSCs in the pre-niche could be a new therapeutic or preventive strategy for CRC metastasis [28]. Lin et al. discovered that an S1PR1-STAT3-IL6-MDSCs axis may be involved in promoting CRC liver metastasis and that MDSCs can form a “pre-metastasis niche” for CRLM. In tumor cells and the tumor microenvironment, sphingosine 1-phosphate receptor 1 (S1PR1) was involved in the continuous activation of signal transducer and activator transcription 3 (STAT3). Increased S1PR1 protein expression activated STAT3 and increased IL-6 expression; IL-6, in turn, activated STAT3 and recruited MDSCs to the liver’s pre-niche. STAT3 was a key transcriptional effector of the signaling pathway dependent on S1PR1, promoting CRC cell growth and liver metastasis. As a result, inhibiting STAT3/S1PR1 signaling in CRC cell can reduce IL-6 expression and myeloid cells infiltration, thereby preventing metastasis [10]. Exosomes play a critical role in the formation of the pre-metastasis niche. Zhao et al. discovered that miR-934 was overexpressed in CRC patients with liver metastasis and was linked to a poor prognosis. miR-934 in exosomes of primary CRC cells was transferred to macrophages, where it induced polarization of M2 macrophages by down-regulating the expression of PTEN and activating the PI3K/AKT signaling pathway. Notably, CXCL13 secretion by polarized M2 macrophages can trigger the CXCL13/CXCR5/NFB/p65/miR-934 positive feedback loop in metastatic CRC tumor cells. Crosstalk between CRC cells and tumor-associated macrophages (TAM) promotes CRLM and induces pre-metastasis niche formation, suggesting a potential treatment strategy for CRLM [27]. Kupffer cells (KCs) were also shown to be involved in the pre-niche formation process of CRLM, and the hypoxic microenvironment induced the release of exosomal miR-135a-5p from primary CRC lesions. KCs phagocytosed exosomes of miR-135a-5p in the blood circulation and transferred to the liver, promoting CRC liver metastasis [30]. The exosomal protein ANGPTL1 released by CRC tumors was mainly taken up by KCs. ANGPTL1 down-regulated the expression of MMP9 and impeded vascular leakiness in the pre-niche site to alleviate CRC liver metastasis. [29]

#### CAFs and molecular mechanisms related to liver metastasis of CRC

The tumor microenvironment is made up of tumor stroma and tumor cells, and their interaction promotes cancer growth and metastasis [95]. Cancer-associated fibroblasts (CAFs) have been shown to interact with cancer cells to promote tumor progression. Endoglin is highly expressed on endothelial cells and is required for vascular development, and it is also specifically expressed on the surface of other stromal cells, such as CAFs. The bone morphogenetic protein (BMP)-9 binded to endoglin on CAFs, causing Smad1 phosphorylation and promoting CAFs invasion. TRC105, a neutralizing antibody, can inhibit Smad1 phosphorylation by preventing BMP-9 binding to endoglin, resulting in inhibition of CAFs invasion in vitro and reducing the metastatic spread of CRC cells in the liver, indicating CAFs are also potential therapeutic targets [96]. Furthermore, CAFs also contribute to the EMT, stemness, and metastasis processes by secreting exosomes [97]. J. L. Hu and colleagues discovered that miR-92a-3p-containing exosomes secreted by CAFs in the tumor microenvironment were important mediators of communication between CRC tumor cells and stromal cells. CAFs-exosomal miR-92a-3p was delivered to tumor cells to increase its amount in the cytoplasm. miR-92a-3p bound directly to the target genes of FBXW7 and MOAP1, suppressing their expression via the Wnt/- βcatenin signaling pathway and inhibiting CRC cell EMT and metastasis. Using miR-92a-3p or blocking the function of CAFs to secrete exosomes may be an alternative treatment for CRC metastasis [98]. Although CAFs in the tumor microenvironment has a significant impact on CRLM, the origin of CAFs is unknown. Hao-Xiang Tan et al. discovered that in the environment of CRLM, CAFs were induced by SDF-1. SDF-1 was released by liver-specific pericytes and hepatic stellate cells (HSCs). HSCs were activated by tumor-derived CXCR4 and differentiated into CAFs via the SDF-1-CXCR4-TGF-β pathway to promote liver metastasis. By inhibiting CAFs differentiation via the CXCR4/TGF-β1 pathway, a clinical strategy for anti-metastatic therapy may be developed [99].

#### Immune/inflammation response in the tumor microenvironment

The two cores of the tumor microenvironment are immunity and inflammation, but the relationship between the two is unclear. Inflammation promotes tumor development; persistent inflammatory cells and factors can transform the tumor-related inflammatory microenvironment into an immunosuppressive microenvironment, promoting tumor progression and metastasis [100].

#### Inflammation promotes metastasis

Influential microenvironmental factors may act as metastasis initiators in cancer cells, allowing them to spread to distant organs and establish metastasis at a secondary site. The cytoplasmic membrane-bound pattern recognition receptor nucleotide-binding oligomerization domain 1 (NOD1) receptor can recognize microorganisms and mediate inflammation. Under inflammatory stimulation, NOD1 promoted the metastatic phenotype of CRC cells via the p38 MAPK pathway. It enhanced the adhesion and metastasis of CRC cells to the liver sinusoids, as Henry Y. et al. It is demonstrated that NOD1 can be a novel target of inflammation-mediated cancer metastasis [101]. Wenjing et al. investigated a transcription program mediated by the inflammatory environment. GFI1, a six-zinc finger transcription repressor, was significantly down-regulated after CRC cells were treated with LSMCM (LPS-stimulated Monocyte Conditioned Medium) for 24 h, and the TGFβ signaling pathway was responsible for the down-regulation of GFI1. LSMCM activated the GFI1-STAT3-EP2 signaling pathway, promoting EMT, migratory, and invasive behavior in CRC cells, indicating that inflammation promotes metastatic spread [102].

#### Inflammation results in immunosuppression and promotes metastasis

**Fig. 4:**
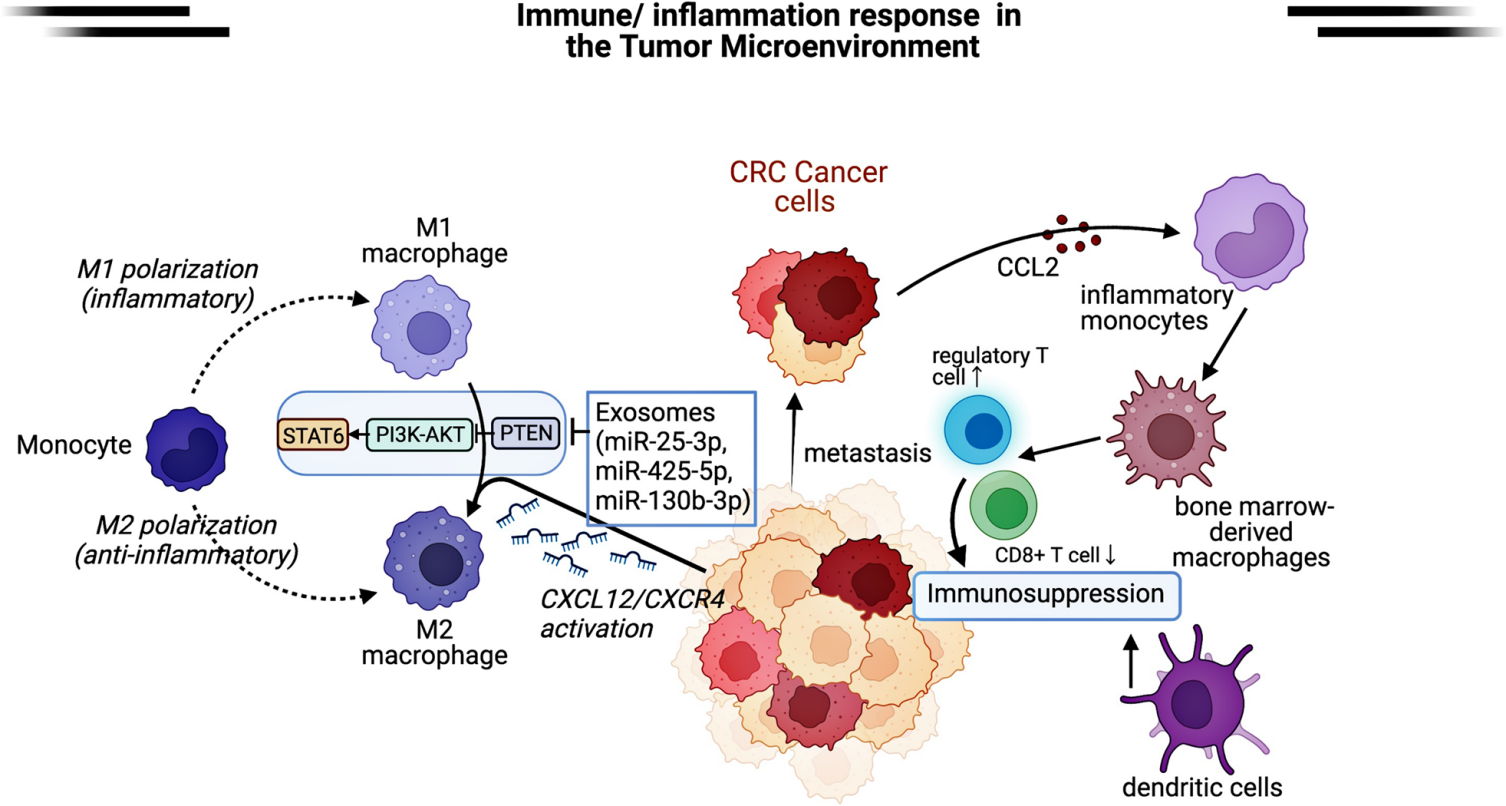
Immune/inflammation response in the tumor Microenvironment. M2 macrophages, inflammatory monocytes, bone marrow-derived macrophages, and dendritic cells cause an immunosuppressive microenvironment. CRC cells with high expression of CXCR4 were activated through the CXCL12/CXCR4 axis and secreted exosomal miRNAs (miR-25-3p, miR-130b-3p and miR-425-5p) to promote M2 polarization of M1 macrophages; CRC tissues with high Expressing CCL2 recruited inflammatory monocytes to metastatic sites through the CCL2/CCR2 chemokine axis promoting immunosuppression; suppressor cells such as myeloid-derived macrophages and dendritic cells were predominant during CRLM process

As the most common immune cells in the tumor microenvironment, macrophages play a critical regulatory role in the progression of inflammation and tumor metastasis. Circulating monocyte precursors are recruited to tumor sites, infiltrating tumors, termed TAMs. Macrophages have diverse phenotypes and functions, and at least two phenotypes of TAMs are involved in tumor promotion. Classically-activated (M1) macrophages secrete pro-inflammatory factors and pronounced anti-tumor activity, but the alternatively-activated (M2) macrophages can suppress the immune system by forming a barrier around invasive cells and establishing a “microenvironment” in which tumor cells can escape immune surveillance and metastasize. The intrinsic plasticity of TAMs controlled the balance between tumor-suppressing and tumor-promoting activities. [54]. Li et al. discovered that Ndrg2 deficiency increases the activation of the NF-κB pathway in TAMs, which regulated TAMs polarization toward the tumor suppressor phenotype (M1). Besides hepatic macrophages, Kupffer cells, a subtype of bone marrow-derived macrophages, are also responsible for inhibiting CRLM [103]. As previously stated, certain secreted factors (such as exosomes) influence distant tumor metastasis by regulating TAM polarization, forming a regulatory loop between tumor cells, TAMs, and the microenvironment [103]. Dong Wang et al. discovered that CXCR4 was overexpressed in CRC tissues and patients with high CXCR4 expression have a higher risk of metastasis. miR-25-3p, miR-130b-3p, and miR-425-5p were highly expressed in CRC cells activated by the CXCL12/CXCR4 axis. These miRNAs secreted by CRC cells were encapsulated in exosomes and delivered to TAMs. TAMs with increased miR-25-3p, miR-130b-3p, and miR-425-5p, promoted their M2 polarization, and PTEN acted as a downstream target to activate the PI3K/Akt signaling pathway. M2 polarization of macrophages enhanced the CRC cells’ metastatic capacity, promoting CRC liver metastasis [104]. The ability of monocytes to be recruited into the tumor microenvironment and differentiate into TAMs has intrigued researchers. According to research, the heterogeneity of myeloid cell phenotype appeared to be selective for tumors and organs, implying that bone marrow cells with different markers drive metastasis to other sites. A study, for example, confirmed that CRC expresses CCL2 to mobilize inflammatory monocytes (IM) from the bone marrow via the CCL2/CCR2 chemokine axis. CCL2 were highly expressed in metastatic CRC liver tumors compared with normal liver tissue, and CCL2 mediated bone marrow-derived circulating CCR2 + IM (CD11b + , CD14 + , CCR2 +) migration to the site of liver metastasis in CRC patients, where they differentiated into immunosuppressive TAMs (CD115 + , CD14 + , CD68 +) and supported metastatic tumor growth. As a result, the ability of CCR2 + IM to decrease CD8 + T cells and increase regulatory T-cells (CD4 + , FoxP3 + , CD25 +) may be a potential mechanism for its promotion of tumor growth at the metastatic site. In addition, it has recently been discovered that blocking CCR2 produced an anti-tumor immune response at the metastatic site, providing a new target for clinical treatment [105]. According to a recent single-cell analysis of autologous samples of liver metastasized CRC, suppressor cells such as dendritic cells (DC3s) and SPP1 + macrophages were predominant in liver metastasis, forming a more immunosuppressive environment, resulting in the pro-metastasis effect [106]. In addition, one study found that the metastatic liver microenvironment had a marked spatial reprogramming effect on immunosuppressive cells such as MRC1 + CCL18 + M2-like macrophages. By defining the immune landscape of CRLM using single-cell RNA-seq and spatial transcriptomics, MRC1 + CCL18 + macrophages exhibited the highest metabolic activity at metastatic sites. This immune reprogramming may be induced by chemotherapy (Fig. 4) [107].

## Biomarkers related to early detection, therapeutic efficacy prediction, and recurrence risk of liver metastasis from CRC

The unpredictability of distant metastasis limits treatment options as well. Thus, identifying biomarkers for liver metastasis and treating CRC will benefit the patients [108]. In Table 2, all therapeutic targets mentioned are summarized. Table 3 summarizes the potential diagnostic, predictive, or prognostic biomarkers for CRLM and their strengths and limitations are summarized.

**Table 2:** Potential therapeutic targets for CRLM

Therapeutic target	Deregulation	Signaling pathway	Role in the metastasis process	Functions	References
Non-coding RNA					
circNSUN2	Up	CircNSUN2/HMGA2/CXCR4	Forms the circNSUN2/IGF2BP2/HMGA2 RNA–protein ternary complex	Enhances the stability of HMGA2 mRNA to promote CRC cell aggressiveness	[60]
miR-424	Down		Specifically targets FGFR1 to inhibit its expression		[71]
miR-30b	Up		PDGFRB inhibits miR-30b expression		[72]
circPPP1R12A	Up	Hippo-YAP signaling pathway	CircPPP1R12A encodes a functional protein circPPP1R12A-73aa that activates the Hippo-YAP signaling pathway	Promotes the proliferation, migration, and invasion of colon cancer cells	[62]
miR-934	Up	miR-934 downregulates PTEN expression and activates PI3K/AKT signaling pathway in TAMs	Induces polarization of M2 macrophages, polarized M2 releases CXCL13 to activate the CXCL13/CXCR5/NFκB/p65/miR-934 positive feedback loop in metastatic CRC cells	Forms an inflammatory microenvironment to foster CRLM	[27]
miR-92a-3p	Up	Wnt/β-catenin signaling pathway	miR-92a-3p targets FBXW7 and MOAP1, suppressing their expression	Enhances stemness, EMT, and metastasis of CRC cells	[98]
LncRNA UICLM	Up		UICLM down-regulates ZEB2 expression	Promotes liver metastasis of CRC cells	[137]
Chromosome					
chromosome 5	Amplification			Upregulates MMPs, promotes the EMT and invasion of colon cancer cells	[52]
Protein					
S1PR1	Up	S1PR1–STAT3–IL-6–MDSCs axis	The mutual activation loop between S1PR1 and STAT3 increases IL-6 expression and recruits MDSCs into liver pre-niches	Promotes the proliferation, migration, and invasion	[10]
c-met	Up		c-met activation triggers a series of signaling cascades	Promotes the proliferation and prevents the apoptosis	[61]
FBX8	Up		FBX8 degrades HIF-1α, CDK4, and C-myc	Inhibits cell cycle progression, proliferation, angiogenesis, and metastasis	[90]
CXCR4	Up	The CXCL12/CXCR4 axis activates CRC cells to promote the secretion of exosomal miR-25-3p, miR-130b-3p, and miR-425-5p, which are delivered to TAMs to induce M2 differentiation of TAMs	M2 macrophages cause immune suppression of the microenvironment	Enables CRC cells to escape immune surveillance	[99]
Binding oligomerization domain 1 (NOD1) receptor	Up	p38 MAPK signaling pathway	C12-mediated NOD1 activation interacts with tumor cells and collagens and fibronectins of ECM	Increases ECM adhesion and migration	[101]
transcriptional repressor GFI1	Down	GFI1-STAT3- EP2 signaling pathway in CRC cells	Down-regulated GFI1 increases fibronectin, and vimentin, decrease E-cadherin	Inhibits the EMT, migration, and invasion	[102]
METTL14	Up		METTL1 inhibits the expression of lncRNA XIST	Inhibits the proliferation and invasion of colorectal cancer cells	[121]
Cell type					
Lgr5 + CSC			Self-renew, asymmetrical division	Drives metastasis initiation	[81]
Lgr5- CSC			Replenishes the damaged CSC pool	Promotes dissemination, cell escape, metastasis colonization	[81]
CD44v6 + CSC		OPN,TGF-β and SDF-1 produced by CAFs activate CD44v6 + CSC via Wnt/β-catenin pathway in CD44v6 + CSCs	Self-renewal	Promotes migration and metastasis colonization	[82]
BMI-1 + CSC		Autophagy-lysosome pathway in BMI-1 + CSCs	Self-renewal	Promotes proliferation and metastasis	[86]
CXCR2 + MDSC		CXCL1–-CXCR2–-MDSCs axis	CXCL1 in the pre-metastatic liver recruits CXCR2-expressing MDSCs into the pre-metastatic liver	Promotes cancer cell survival, metastatic tumor formation, and growth	[28]
Endoglin-expressing CAFs		TGF-β/BMP signaling pathway in CAFs	interacts with tumor cells	Promotes CAFs invasion and colonization	[96]
CCR2 + CD11b/ Gr1 mid myeloid cells			CCL2 mediates CCR2 + inflammatory monocytes differentiate into immunosuppressive TAMs (CD115 + , CD14 + , CD68 +)	Promotes cancer cells extravasation and growth	[105]
MRC1 + CCL18 + M2-like macrophages			Organ-specifically distributed in the liver exhibits a metabolically high-energy phenotype and is sensitive to adjuvant therapy	Induces suppressive immune microenvironment to promote cancer cells growth and metastasis	[120]

**Table 3:** Potential diagnostic, predictive or prognostic biomarkers for CRLM

	Biomarker	Clinical practice	Strengths	Limitations	Refs.
Mutated genes	BRAF, KRAS, TP53, PIK3CA, and members of the SMAD family	Potential prognostic predictors for overall survival after liver surgery for CRLM	Expression is highly consistent between primary and metastatic tumors, and molecular assays can be performed on resected samples of primary tumors	Biomarkers cannot predict primary tumor nodal status, limiting their use as tools to guide surgical patterns	[110]
Differentiated expressed genes	ERCC1	Potential predictive biomarkers for the response rate to platinum-based chemotherapy in patients with CRLM	A high level of reproducible predictive markers in pathology practice benefits ERCC1 as a predictive biomarker for platinum-based chemotherapy	The interobserver agreement is low; ERCC1 IHC expression in primary tumors is low in association with metastatic liver events, implying the need to use tissue from actual tumor burden to evaluate ERCC1 expression; and treatment alters ERCC1 expression	[112]
	EMX2	Down-regulated EMX2 is a strong predictor of CRLM	EMX2 has predictive value as a prognostic factor in CRC and may have a functional role in metastatic spread, so that EMX2 inhibition may be a promising therapeutic strategy	Limited power for statistical inference due to the small sample size; whether it is a driver of metastasis or a coincidence of tumor progression remains to be further explored; Adenoviral vectors serve as delivery systems to restore EMX2, but adenoviral vector therapeutic strategies for cancer patients remain challenging	[113]
Chromosome	Chromosome 4	Chromosome 4 deletion can be a potential predictive biomarker for primary tumor progression, long-term survival, and recurrence after complete metastases resection	It serves as a prognostic biomarker to provide evidence for the decision to use adjuvant chemotherapy in patients with CRLM after metastases resection, early identification of patients with good prognosis who do not require further adjuvant therapy, and reduced side effects	The small sample size for preliminary studies	[114]
miRNAs	Serum exosomal miR-122	As a diagnostic marker of CRC with LM	Serum exosomal miR-122 has high specificity and can distinguish CRC patients with LM from CRC without LM	Small sample size	[116]
	Exosomal miR-21, miR-203, and miR-210	Non-invasive prognostic biomarker for predicting liver metastases	Circulating molecules are stable, reproducible, and consistent across individuals. They can be used as predictive biomarkers to distinguish liver metastatic CRC from other metastatic or non-metastatic CRC	The expression of miRNAs varies widely among tumor populations; levels of circulating miRNAs can be significantly altered by hemolysis, and techniques for sample storage, RNA extraction, and miRNA quantification need to be improved	[119]
LncRNA	LncRNA Yiya	Independent prognostic biomarkers for CRLM	Colorectal liver metastases generally rely on portal vein drainage, and Yiya is associated with hematogenous spread, which has considerable value in early prediction and timely clinical intervention		[120]
CircRNA	CircRNA_0001178 and circRNA_0000826	Potential diagnostic biomarkers for CRLM	CircRNAs are tissue-specific and, therefore, suitable as cancer biomarkers		[117]
Others	Apoptotic circulating tumor cells	Potential predictive biomarkers for CRLM	Apoptotic CTCs, CTC fragments but not CTCs, are useful liquid biopsy markers associated with worse overall and progression-free survival, especially in metastatic liver disease	The heterogeneity of patient populations, including different prior treatments and disease genetics, must also be considered	[123]
	Leptin and the activity of chitinase	Potential predictive biomarkers for CRC liver metastasis	Serum chitinase activity is an independent risk factor for predicting liver metastasis, and the effect is superior to traditional CEA	Dividing subjects into groups makes the amount of data in each group smaller	[124]

### Mutated genes as biomarkers

In recent years, mutated and differentially expressed genes have been identified as potential predictive and prognostic biomarkers for CRLM. The incidence of BRAF mutation in metastatic CRC is less than 10%, but BRAF V600E mutation is thought to be a relevant therapeutic target for metastatic CRC management. Although the data showed that CRC patients with KRAS mutations had a higher risk of liver metastasis, the difference was not statistically significant, and the risk of liver metastasis in patients with BRAF mutations was lower than that in patients with Wt BRAF. As a result, there was no clear link between KRAS and BRAF mutations and liver metastasis in patients with CRC [109]. A multivariate Cox regression model has been used in studies to predict genes with potential prognostic significance after CRLM resection. Mutations in the TP53, KRAS, PIK3CA, BRAF, and SMAD families have been identified as prognostic markers for overall survival after CRLM liver surgery. The SMAD family of genes, which included SMAD2, SMAD3, and SMAD4, had the best predictive value for poor tumor outcomes [110].

### Differentiated expressed molecules as biomarkers

ERCC1 may serve as a predictive biomarker for the effectiveness of chemotherapy. The expression of ERCC1 was primarily examined in the relationship between CRC and liver metastasis. There was no clear evidence of a link between ERCC1 expression and concurrent/metachronous liver metastasis, and no evidence of a link between the primary tumor and liver metastasis was found. A study examines ERCC1 protein in primary colorectal cancer and corresponding liver metastases. Good reproducibility of IHC expression benefited ERCC1 as a predictive biomarker for platinum-based chemotherapy. However, no consistency has been observed when comparing IHC expression in primary tumors and matched liver metastases. It means ERCC1 expression of liver metastases can not be assessed in archived material (primary tumor biopsies), implying the need to use tissue from actual tumor burden to evaluate ERCC1 expression [111, 112]. The EMX2 gene belongs to the family of homeobox genes. The Cox proportional hazard model was used to examine the distant metastasis rate of EMX2 overexpression tumor patients and EMX2 low expression tumor patients. The results showed that there is a significant correlation between tumor EMX2 expression down-regulation and CRLM [113].

### chromosome 4 deletion

A study published in 2013 used array-based comparative genomic hybridization (aCGH) to examine genetic changes in metastatic site samples from two groups of CRC patients with liver metastases who had survival rates of less than five years and more than five years. Through survival analysis, metastatic tissue samples with whole chromosome 4 loss indicated a higher survival rate in the patients, implying that the absence of chromosome 4 may reduce the risk of recurrence of CRLM [114].

### Non-coding RNAs as biomarkers

Non-coding RNAs regulate the development and metastasis of CRC and have a significant effect on cancer biology. Moreover, the primary tumors release a variety of exosomes containing various LncRNAs to promote the formation of the anterior liver niche, molecules released by exosomes can be detected by liquid biopsy, and LncRNAs can be used as an effective biomarker to predict early metastasis, especially micrometastases that cannot be detected by pathological methods [115]. Li Sun et al. discovered, for example, that miR-122, packaged into exosomes, was significantly overexpressed in CRC patients with liver metastasis compared with CRC patients without liver metastasis [116]. Compared with primary CRC tissues, circRNA_0001178 and circRNA_0000826 were significantly upregulated in CRC liver metastasis tissues. And it was found that circ102049 was highly expressed in primary CRC tumors with liver metastasis and closely correlated with the prognosis of CRC patients. These non-coding RNAs may serve as potential diagnostic biomarkers for CRC liver metastasis [117, 118].

### miRNAs

miRNA is a type of short single-stranded endogenous non-coding RNA that can bind to the 3'-untranslated region (3'-UTR) of mRNA to regulate transcription, resulting in translational inhibition. When miRNA expression in the plasma of CRC patients with and without liver metastases was compared, miR-21, miR-203, and miR-210 were significantly dysregulated in patients with liver metastatic CRC, indicating their roles as early detection biomarkers. Significantly downregulated miR-19a, miR-203, and miR-21 have been shown to have the ability to predict patients at high risk of recurrence [119].

### LncRNAs

LncRNAs, typically > 200 bp in length, are natural prognostic biomarkers. Yiya and GAS5 expression were upregulated at liver metastasis compared with the primary CRC, and CRC patients with high expression of Yiya and GAS5 had a higher risk of liver metastasis. Yiya’s expression was found to be an independent risk factor for liver metastasis in early-stage patients using Cox proportional-risk regression analysis, suggesting that it could be the most potential predictive biomarker for liver metastasis [120]. The most crucial catalytic enzyme for mRNA methylation modification has been identified as METTL4, a major m^6^A methyltransferase. METTL14’s downstream target was lncRNA XIST, according to Xiao Yang et al. Down-regulation of oncogenic lncRNA XIST via METTL14 inhibited CRC cell metastasis and growth; thus, METTL14 may be a promising therapeutic target [121].

### CircRNA

Hanchen Xu et al. discovered a difference in circRNA expression between tissue samples from CRC patients with and without liver metastasis. It was discovered that circRNA_0001178 and circRNA_0000826 might be potential diagnostic biomarkers for CRLM by constructing a circRNA-miRNA-mRNA network [117].

### Other prognostic biomarkers of colorectal cancer liver metastasis

Some small-scale cohort studies have discovered that various types of circulating molecules can be used as potential biomarkers for prognostic prediction of CRLM. However, their actual clinical applications were still minimal [122]. There was evidence that apoptotic circulating tumor cell (CTC) was a possible prognostic marker for CRC metastasis to the liver or other metastases. Among the 17 patients with metastatic CRC included in the study, 6/11 had apoptotic CTCs in the liver, and 1/9 had apoptotic CTCs in other metastatic sites [123]. The expression of leptin and the chitinase activity can predict the ability of CRC to metastasize. Leptin expression was significantly higher in metastatic CRC tissue than in primary tumors without liver metastasis, indicating that increased leptin expression was associated with a higher risk of liver metastasis. Chitinase is an inflammatory biomarker that causes inflammatory factors to be produced. According to the findings of multi-factor regression analysis, serum chitinase activity was an independent risk factor for predicting liver metastasis, and the power was superior to traditional CEA. This conclusion was consistent with the view that inflammation promotes cancer metastasis [124].

## Challenges and perspectives

CRC liver spread is difficult to detect very early, with limited treatment options and a poor prognosis, resulting in median survival of about six months for CRLM patients [125]. There was no clear stratification for patients with resectable metastatic tumors. Fong clinical risk scores included multiple variables that stratify patients with distant metastasis into high-risk (5-year OS: 47%) and low-risk (5-year OS: 24%) groups, but they lack validation from a large cohort of neoadjuvant chemotherapy patients, limiting their accuracy [126, 127]. The development of adjuvant systemic therapy has significantly improved the clinical outcome of patients with stage IV CRLM [7, 128]. CRC heterogeneity presents significant challenges in disease diagnosis and treatment; genetic/epigenetic alterations may be a driving force at the genome level, giving rise to subclones with potential metastatic spread. Furthermore, the non-genetic diversity of phenotypic and behavioral states of cancer cell clones has been proposed as a source of intra-tumor heterogeneity, which is influenced by changing microenvironments [129]. Although many studies have identified one or more genes, molecules, and pathways related to metastasis, their influence on phenotypes of metastatic advantages has yet to be investigated.

CRC metastasis may occur at the stage of tumorigenesis. In recent years, genomic analysis in the difference between primary tumors and metastatic sites has challenged the notion that CRC metastasis is an early event. In addition to the evidence-based genomic analysis, the “seed-soil” theory and the formation of pre-niches also suggest that metastasis occurs at an early stage and that specific molecules secreted by the primary tumor predispose to the occurrence of CRC in the liver. “Parallel evolution” discovered that the genetic divergence between the primary tumor and the metastatic site is greater than that of the “linear evolution model”, implying that in CRC, some metastatic clones leave the primary site at an early stage and evolve further within the metastases [33]. The central question is the mechanisms underlying the formation of metastatic clones, like whether metastatic capacity is an advantage that has been selected during tumor evolution in the primary tumor or just an unselected, incidental consequence of tumor progression [43]. Because only a few “fit” subclones among all clones can survive and give rise to metastasis in a distant organ, several cancer initiation evolution models have been proposed to explain metastatic progression, and the feasibility of these models in explaining the development of metastasis is still being explored [122]. Tumor initiation is thought to be driven by the sequential stepwise acquisition of genetic alternations involving driver gene mutations in the classical “linear progression model” (Note: this should be distinguished from the “linear progression model” in metastatic evolution). A series of selective sweeps can fixed new driver mutations leading to clones expanding and providing strong selective advantages. The extent to which the selection effect promotes clonal diversity and generates dominant clones, on the other hand, is still debatable. The “Neutral evolution model,” which contradicts classic Darwinian evolution, hypothesizes that “random drift, rather than non-random selection based on reproductive fitness, results in the generation of new clones.” Rather than the sequential clonal evolution (“linear progression model”), the “Big Bang” model proposes that heterogeneous cell populations with potential metastasis advantages are simply “incidental byproducts” of the primary tumor formation process. Clonal selection has no effect on tumor progression, and these intermixed subclones have similar fitness and grow at the same rate [47, 130]. It has been discovered that the “Big Bang” model and its subsequent “Neutral evolution model” best explain the emergence of metastatic clones in CRC.

The role of molecular genetic changes in metastatic evolution is still largely unknown. Here, we found that epigenetic changes were more likely than genetic changes to be the direct driver of metastasis, and certain gene mutations were associated with specific metastasis patterns. However, studies at the genome level have demonstrated that metastatic CRC is of monoclonal and polyclonal origin and follows both monophyletic and polyphyletic seeding patterns. By identifying critical genes in metastatic evolution, it may be possible to improve the ability to determine whether CRC has metastasized. Recent research on the genome landscape of patients with metastatic CRC has revealed a link between mutant genes and metastasis patterns. S-C evolutionary pattern was associated with KRAS, SYNE1, CACNA1H, PCLO, FBXL2, and DNAH11 [50]. However, no universal metastasis-specific driver genes have been identified in CRC comparative genome studies. The epigenetic alterations associated with CRLM observed in the studies primarily included DNA methylation, non-coding gene regulation, and chromosome remodeling [40, 131].

Clinical evidence indicates that reasonable combination strategies are essential in the clinical treatment and efficacy of CRC liver metastases [132]. The tumor immune microenvironment influences the progression of malignant tumors. For example, immune checkpoint proteins (PD-L1, CTLA-4, TIM3), the main targets of immunotherapy, are present in exosomes and can promote tumor progression and metastasis [133, 134]. Some immune infiltrations can suppress adaptive immunity. The liver- metastasized lesion contains a complex tumor microenvironment that is easier to produce an immunosuppressive microenvironment than other organs to promote tumor metastasis. As a result, the immune microenvironment is regarded as a promising therapeutic target for CRLM [135]. For example, combining immune checkpoint blocking therapy with conventional therapy opens up new treatment options. According to one study, increased circulating fms-related tyrosine kinase 3 ligand (FLT3LG) caused by neoadjuvant cytotoxic treatment in rectal cancer patients activated the anti-tumor immune response. Functionally active adaptive immune cells may eliminate microscopic tumor cells, lowering the risk of tumor metastasis [136]. The combination of IL-12 gene therapy and chemotherapy has shown the ability to transform the metastatic microenvironment into a more immunogenic phenotype by reducing Treg, MDSC, and M2 macrophages [132].

## Conclusions

This review summarized the main perspectives related to metastasis, especially focusing on the factors influencing organ-specific metastasis. Beginning with general metastasis principles to understand the process of metastasis, we summarize and integrate the hypothesis of the metastatic evolution patterns (including origin of metastatic cloning and the pattern of metastatic seeding), genetic/epigenetic changes related to CRLM, molecular and cellular mechanisms, and biomarkers providing ideas for challenging metastatic CRC. The phenotypic change of CSCs is also one of the essential intrinsic factors for CRLM, which is a prerequisite for metastasis formation. For extrinsic factors of CRLM, cell/molecular interactions between tumor cells and the tumor microenvironment have been shown to mediate metastasis. Among them, CAFs are critical cellular components that induce CRLM, and the establishment of an immunosuppressive microenvironment has been established in the metastatic site. The relationship between inflammation and immune response needs to be further explored. Several central questions remain to be clarified: (1) Whether and what kind of the evidence based on the genome level can predict the potential of CRC to develop liver metastases in the early stage and predict the efficacy of immunotherapy, to provide a reference for clinical treatment strategies? (2) The dynamic evolution of cancer stem cell phenotypes is a prerequisite for CRLM. How can it be an effective therapeutic target or a biomarker for early detection? (3) Since macrophages and CAFs play an essential role in CRLM, whether they affect the organ tropism of metastasis? What is possible to detect CRLM early by detecting cell phenotypes in the tumor microenvironment? And whether can we predict a state of suppressive immune microenvironment for a tumor? Exploration of these issues will provide helpful information for early detection and appropriate treatment strategies for CRLM. Finally, we summarize the current therapeutic targets for CRLM, as well as diagnostic/prognostic biomarkers, and analyze the strengths and limitations of these methods, hoping to provide valuable clues for the diagnosis and treatment of CRLM.

## Data Availability

All data included in this study are available upon request by contact with the corresponding author.

## Abbreviations

CRC: Colorectal cancer
CRLM: Colorectal cancer liver metastasis
SNVs: Single nucleotide variations
sSNVs: Somatic single nucleotide variations
LM: Liver metastasis
CCF: Cancer cell fractions
EMT: Epithelial-mesenchymal transition
CNVs: Copy number variations
MMPs: Matrix metalloproteinases
TKI: Tyrosine kinase inhibitor
C–C: Clonal- Clonal
S-C: Subclonal- Clonal
0-C: None- Clonal
HLA: Human leukocytes antigens
LOH: Loss of heterozygosity
CGI: CpG island
HGF: Hepatic growth factor
m^6^A: N6-methyladenosine
Circ RNAs: Circular RNAs
LncRNAs: Long non-coding RNAs
CYTOR: Cytoskeleton regulator RNA
CSC: Cancer stem cell
MCSCs: Migrating CSCs
TA: Transient amplification
TICs: Tumor- initiating cells
LGR5: LT-TICs: long term tumor- initiating cells G-protein-coupled receptor 5
ISC: Intestinal stem cell
MDSCs: Myeloid- derived suppressor cells
S1PR1: Sphingosine 1-phosphate receptor 1
STAT3: Signal transducer and activator of transcription 3
CAFs: Cancer-related fibroblasts
BMP: Bone morphogenetic protein
NOD: Nucleotide-binding oligomerization domain 1
LSMCM: LPS-stimulated Monocyte Conditioned Medium
IM: Inflammatory monocytes
DC: Dendritic cell
aCGH: Array-based comparative genomic hybridization
3'-UTR: 3'-Untranslated region
lncRNAs: Long non-coding RNAs
CTC: Circulating tumor cell
circRNAs: Circular RNAs
pre-Micro RNA: Pre-miRNAs
FLT3LG: Fms-related tyrosine kinase 3 ligand
